# Hydrogen peroxide depolarizes mitochondria and inhibits IP_3_-evoked Ca^2+^ release in the endothelium of intact arteries

**DOI:** 10.1016/j.ceca.2019.102108

**Published:** 2019-12

**Authors:** Xun Zhang, Matthew D. Lee, Calum Wilson, John G. McCarron

**Affiliations:** Strathclyde Institute of Pharmacy and Biomedical Sciences, University of Strathclyde, 161 Cathedral Street, Glasgow G4 0RE, UK

**Keywords:** Vascular, Endothelium, Calcium, Hydrogen peroxide, Free radical, Inositol 1,4,5‐trisphosphate

## Abstract

Hydrogen peroxide (H_2_O_2_) is a mitochondrial-derived reactive oxygen species (ROS) that regulates vascular signalling transduction, vasocontraction and vasodilation. Although the physiological role of ROS in endothelial cells is acknowledged, the mechanisms underlying H_2_O_2_ regulation of signalling in native, fully-differentiated endothelial cells is unresolved. In the present study, the effects of H_2_O_2_ on Ca^2+^ signalling were investigated in the endothelium of intact rat mesenteric arteries. Spontaneous local Ca^2+^ signals and acetylcholine evoked Ca^2+^ increases were inhibited by H_2_O_2_. H_2_O_2_ inhibition of acetylcholine-evoked Ca^2+^ signals was reversed by catalase. H_2_O_2_ exerts its inhibition on the IP_3_ receptor as Ca^2+^ release evoked by photolysis of caged IP_3_ was supressed by H_2_O_2_. H_2_O_2_ suppression of IP_3_-evoked Ca^2+^ signalling may be mediated by mitochondria. H_2_O_2_ depolarized mitochondria membrane potential. Acetylcholine-evoked Ca^2+^ release was inhibited by depolarisation of the mitochondrial membrane potential by the uncoupler carbonyl cyanide 3-chlorophenylhydrazone (CCCP) or complex 1 inhibitor, rotenone. We propose that the suppression of IP_3_-evoked Ca^2+^ release by H_2_O_2_ arises from the decrease in mitochondrial membrane potential. These results suggest that mitochondria may protect themselves against Ca^2+^ overload during IP_3_-linked Ca^2+^ signals by a H_2_O_2_ mediated negative feedback depolarization of the organelle and inhibition of IP_3_-evoked Ca^2+^ release.

## Highlights

• H_2_O_2_ is produced by several cell processes including mitochondria and may act as an intracellular messenger and cell-cell signalling molecule.
• Spontaneous local Ca^2+^ signals and IP_3_-evoked Ca^2+^ increases were inhibited by H_2_O_2_.
• H_2_O_2_ suppression of IP_3_-evoked Ca^2+^ signalling may be mediated by mitochondria via a decrease in the mitochondrial membrane potential.
• H_2_O_2_-induced mitochondrial depolarization and inhibition of IP_3_-evoked Ca^2+^ release, may protect mitochondria from Ca^2+^ overload during IP_3_-linked Ca^2+^ signals.

## Graphical abstract

**Figure:**
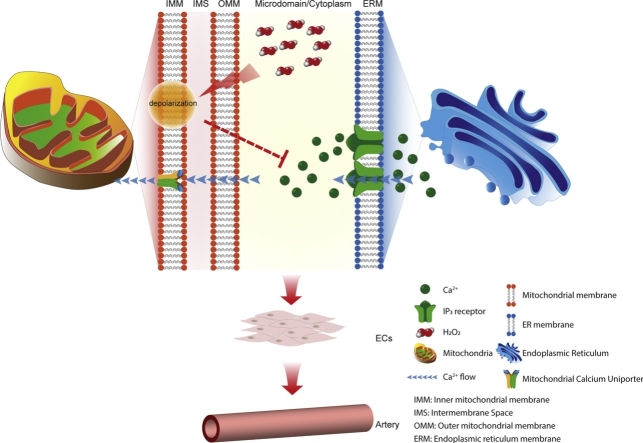

## 1 Introduction

The endothelium is the single layer of cells that lines the entire cardiovascular system and it is exposed constantly to a wide range of mechanical and chemical stimuli. The endothelium responds to these stimuli by releasing Ca^2+^-dependent vasoactive factors that include nitric oxide, prostacyclin, endothelium-derived contracting factors, von Willebrand factor, tissue plasminogen activator and endothelial derived hyperpolarising factor (1, 2). These vasoactive factors allow the endothelium to regulate almost all cardiovascular activities including vascular tone, immune responses, angiogenesis and vascular remodelling [1].

There is accumulating evidence that reactive oxygen species (ROS) also regulates endothelial function. ROS modulates endothelial cell growth, proliferation, endothelium-dependent relaxation, cytoskeletal reorganization, inflammatory responses and endothelium-regulated vascular remodelling. Among various ROS, hydrogen peroxide (H_2_O_2_) fulfils the prerequisites for serving as an intracellular messenger and acting as a cell-cell signalling molecule. H_2_O_2_ is a small and non-polar molecule produced by several cell processes that include mitochondria and NADPH oxidase [17,29,76]. During mitochondrial ATP production, the electron transport chain leaks electrons from complexes I and III resulting in the formation of superoxide anion radical ($O_{2^{-}}$) [11]. $O_{2^{-}}$ generates H_2_O_2_ spontaneously, or by the activity of superoxide dismutases. Although $O_{2^{-}}$ is not membrane permeable, H_2_O_2_ can diffuse across biological membranes or may cross membrane boundaries via channels like aquaporins [8] to regulate physiological and pathological cellular processes [3,62,75,85].

Many of the systems that produce H_2_O_2_, such as mitochondria, are modulated by the cytoplasmic Ca^2+^ concentration [6,24,36]. Ca^2+^ released from IP_3_Rs may result in Ca^2+^signals that propagate into the mitochondrial matrix [35,59]. Changes in mitochondrial Ca^2+^ may lead to enhanced ATP synthesis [74] and, as a result, increased ROS production [68]. Conversely, increased H_2_O_2_ generated by mitochondrial activity may modulate Ca^2+^ signalling to exert control on endothelial function. For example, in various cultured cell lines, H_2_O_2_ evokes Ca^2+^ release from the internal Ca^2+^ store [27,37,77].These observations raise the possibility that there may be feedback regulation of mitochondrial ATP production by changes in the cell activity mediated via the cytoplasmic Ca^2+^ concentration. To explore this possibility we measured the effects of H_2_O_2_ on Ca^2+^ signalling in the endothelium in large numbers of endothelial cells in intact blood vessels. We show that H_2_O_2_ depolarises mitochondria and suppresses IP_3_ evoked Ca^2+^ signalling.

## 2 Methods

### 2.1 Animals

All animal husbandry and euthanasia were carried out in accordance with the prior approval of the University of Strathclyde Animal Welfare and Ethical Review Body and under relevant UK Home Office Regulations, [Schedule 1 of the Animals (Scientific Procedures) Act 1986, UK]. Strathclyde BPU is a conventional unit which undertakes FELASA quarterly heath monitoring. Male Sprague-Dawley rats (10–12 weeks old), from an in-house colony, were used in the study. Animals were housed 3 per cage (RC2F cages, North Kent Plastics Company, UK), provided with enrichment (aspen wood chew sticks and hanging huts), nesting material (Sizzle nest, LBS Technology, UK), and fresh water and chow (RM1, Special Diet Services, UK) were available *ad libitum*. Room temperature was 19–23 °C (set point 21 °C), humidity was 45–65 %, and a 12 h light cycle was used. Rats were euthanatized by intraperitoneal injection of pentobarbital sodium (200 mg/kg, Pentaject, Merial Animal Health Ltd, UK).

### 2.2 Endothelial Ca^2+^ imaging

First order mesenteric arteries were isolated, placed into a physiological saline solution (PSS), cleaned of adherent fat and then used immediately. Each artery was then cut open and pinned flat on a sylgard block, with endothelial cells facing upward (*en face* preparation). The endothelium was then loaded with acetoxymethyl ester form of the Ca^2+^ indicator, Cal-520 (5 μM) and 0.02% pluronic F-127 in DMSO, for 30 min at 37 °C [55,[79], [80], [81]]. Following incubation, arteries were gently washed before the Sylgard block was inverted and placed in a custom-made bath chamber. The bottom of the chamber was a 0-thickness glass coverslip and two (0.2 μm diameter) steel pins were set between the coverslip and the block to prevent endothelial cells from contacting the coverslip, and to allow solutions to flow across the endothelium. Ca^2+^ images were acquired at 10 Hz on an inverted fluorescence microscope (TE300, Nikon, Japan) using a 40×, 1.4 NA oil immersion lens and a back-illuminated electron-multiplying charge-coupled device (EMCCD) camera (1024 × 1024 13 μm pixels; iXon 888; Andor, UK). Fluorescence excitation (488 nM wavelength) illumination was provided by a monochromator (Horiba, UK).

### 2.3 Localized flash photolysis

In some experiments, the endothelial Ca^2+^ response to local photolysis of caged IP_3_ was examined. In these experiments, the endothelium was loaded membrane permeant, caged IP_3_ (5 μM) for 30 min at 37 °C. A xenon flash lamp (Rapp Optoelecktronic, Germany) was used to uncage IP_3_ [13,47,80]. The output light was filtered using a UG-5 filter to select ultraviolet light. The light was focused and merged into the excitation light path via a fibre optic bundle and long pass dichroic mirror attached to the lens part of the microscope’s epi-illumination attachment [13,53,58]. The area of the photolysis site (∼80 μm diameter) resulted from the fiber optic diameter and the objective lens magnification (40x).

### 2.4 Imaging endothelial mitochondria

To assess mitochondrial membrane potential, arteries were pinned out in a Sylgard coated chamber designed for use on an upright microscope. Mitotracker Green FM (100 nM) was added to the PSS and the endothelium was incubated for 20 min followed by 20 min washing. Tetramethylrhodamine ethyl ester (TMRE) (60 nM) was added to the PSS and the endothelium was incubated 10 min [20,21,80]. TMRE (60 nm) was subsequently present in all perfusion solutions. Minimal photobleaching of TMRE was observed over the 5 min recording periods used. TMRE and Mitotracker Green images (10 Hz) were acquired on an upright microscope (Eclipse FN1; Nikon, Japan) equipped with a 60× water immersion objective (1.0 numerical aperture) and an EMCCD camera (iXon 888; Andor, UK).

### 2.5 Experimental protocols

The effect of H_2_O_2_ on basal endothelial Ca^2+^ activity was studied using a non-cumulative concentration response in the same preparation. In these experiments, a Ca^2+^-free PSS was used and H_2_O_2_ added to the Ca^2+^-free perfusate. To prevent depletion of internal Ca^2+^ stores, arteries were incubated in Ca^2+^-containing PSS between each exposure to H_2_O_2_.

The effect of H_2_O_2_ (with or without catalase, 1000 U ml^−1^) on evoked (acetylcholine, ACh; 100 nM) endothelial Ca^2+^ activity was studied in paired experiments. In these experiments, a control response (5-minute recording) to ACh (flowed rate: 1.5 ml min^−1^) was obtained before the tissue was washed for 5 min, and allowed to equilibrate for 10 min. ACh was then applied a second time, together with H_2_O_2_ (with or without catalase), and the responses compared. ACh and H_2_O_2_ (with or without catalase) were applied via separate syringe pumps each at 0.75 ml min^−1^. The effects of various pharmacological interventions on ACh-evoked Ca^2+^ signalling were also studied in paired experiments in which control responses were first obtained and then the endothelium was incubated with each antagonist for 20 min. Following the incubation period, ACh was applied a second time and responses compared to control. Each pharmacological agent was present throughout the second exposure to ACh.

Experiments utilising caged-IP_3_ also used a paired experimental design. An initial response to photolysis was recorded, and the tissue was then rested for 10 min. The endothelium was then incubated with H_2_O_2_ for 20 min before a second response, using the same photolysis location, was obtained.

### 2.6 Data analysis

Automated analysis of endothelial Ca^2+^ imaging recordings was carried out using custom-written Python routines [44,80,81]. In brief, average intensity projections were used to generate regions-of-interest (ROI) around each cell. The Ca^2+^ response of each endothelial cell was then extracted by averaging the fluorescence intensity within each ROI, for each cell and image in the dataset. Each ROI/cell was assigned an identification number so that the response of each cell could be compared within experimental series. Fluorescence signals are expressed as ratios (F/F_0_) of fluorescence counts (F) relative to baseline (control) values before stimulation (F_0_). The baseline (F_0_) was identified automatically as the 100 frame (10 s) period exhibiting the lowest noise prior to the introduction of any agonist. The total number of oscillations, and the amplitude of each oscillation were then extracted for each cell using a zero-crossing peak-detection algorithm [82] for signals exceeding 3 times the standard deviation of baseline noise.

### 2.7 Statistics

All data are presented as mean ± SEM of n biological replicates. Data were analysed using repeated measures one-way ANOVA with Geisser-Greenhouse correction and Dunnett’s multiple comparisons test, or paired *t*-test as appropriate. A p value less than 0.05 was considered statistically significant. All statistical analysis was performed using GraphPad Prism version 6.0 (GraphPad Software, USA) was used to run the statistical analysis.

### 2.8 Reagents and chemicals

The PSS consisted of (in mM):145 NaCl, 2.0 MOPS, 4.7 KCl, 1.3 NaH_2_PO_4_, 5.0 Glucose, 1.17, MgCl, 2.0 CaCl, 0.02 EDTA (pH adjusted to 7.4 with NaOH). In experiments using Ca^2+^ free PSS, CaCl_2_ was replaced with MgCl_2_ on an equimolar basis and EGTA (1 mM) was included. Caged-IP_3_ (caged-IP_3_ 4,5-dimethoxy-2-nitrobenzyl) was obtained from Sichem (Germany). Cal-520 was obtained from Abcam (UK). Pluronic F-127 was obtained from Invitrogen (UK). Mitotracker Green FM was obtained from Invitrogen (UK). All other drugs and chemicals were obtained from Sigma (UK). Stock solutions of ACh, catalase-polyethylene glycol and H_2_O_2_ were prepared by dissolving each chemical in double-distilled, dionized water. 2-aminoethoxydiphenyl borate (2-APB), caged-IP_3_, Cal-520, carbonyl cyanide m-chlorophynyl hydrazine (CCCP), oligomycin, TMRE and Mitotracker Green FM were dissolved in DMSO.

### 2.9 Data availability

All data underpinning this study is available from the authors upon reasonable request.

## 3 Results

**Fig. 1:**
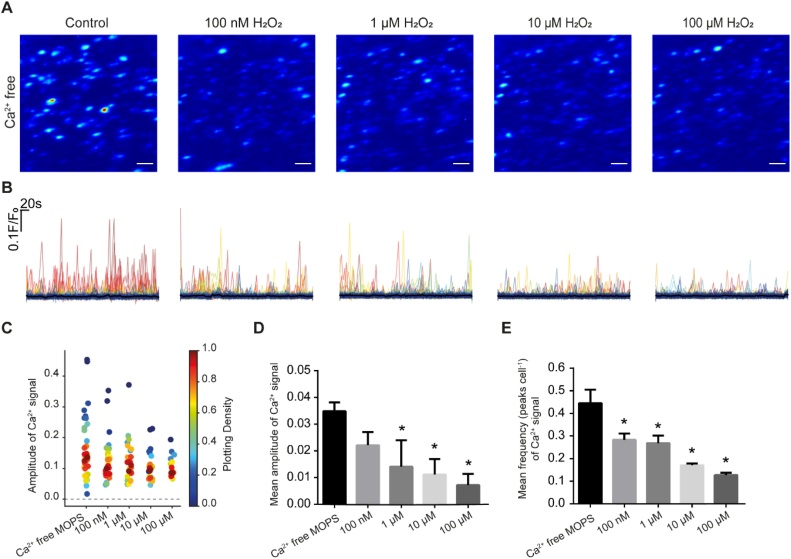
Effect of H_2_O_2_ on spontaneous local Ca^2+^ signals arising from the internal Ca^2+^ stores. (A) Pseudo-colour images of spontaneous Ca^2+^ signals activity (red high, blue low amplitude) over a 5 min period in control (Ca^2+^-free PSS) and with H_2_O_2_ (100 nM to 100 μM). Between each recording, H_2_O_2_ was washed out and Ca^2+^ was restored to the bathing medium (10 min) to permit the store to refill. Scale bar: 20 μM. (B) Ca^2+^ signals measured in ∼200 cells shown in A. (C) Density plot of mean peak value of Ca^2+^ signalling in Ca^2+^ free MOPS and increasing concentrations of H_2_O_2_. Individual data points have been coloured (from blue, low to red, high) according to the density (i.e. occurrence) of particular values. (D) Summary of mean peak value of Ca^2+^ signalling in all cells. (E) Summary of number of Ca^2+^ signalling peaks in all cells *n = 5*, *p<0.05 vs Ca^2+^-free MOPS.

To determine if H_2_O_2_ alters spontaneous Ca^2+^ release from the internal store in intact mesenteric arteries, H_2_O_2_ (100 nM, 1 μM, 10 μM and 100 μM) was applied in a Ca^2+^ free PSS (Fig. 1). Between H_2_O_2_ applications arteries were washed in PSS (containing Ca^2+^) to allow the internal Ca^2+^ stores to refill. As the concentration of H_2_O_2_ increased, spontaneous Ca^2+^ release events decreased (Fig. 1). As spontaneous Ca^2+^ release arises from IP_3_-receptor activity [44,80,81], these results suggest that H_2_O_2_ may suppress Ca^2+^ release from the internal Ca^2+^ store.

**Fig. 2:**
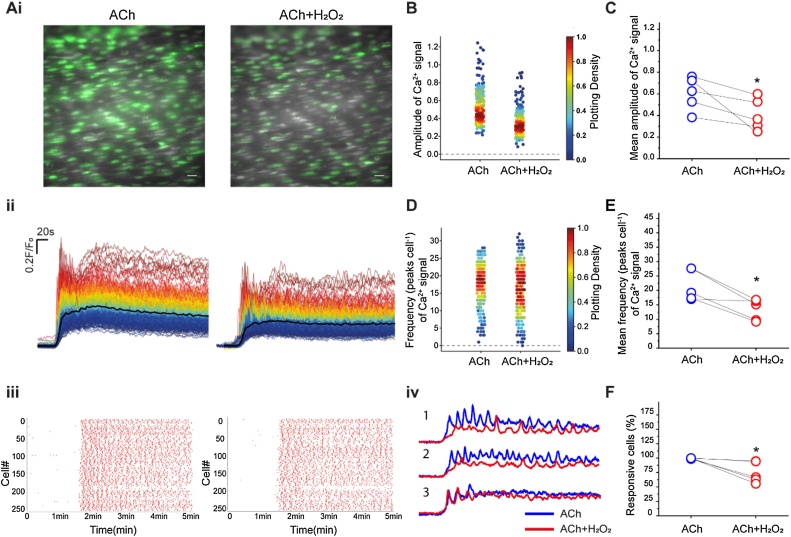
ACh-evoked Ca^2+^ release was suppressed by H_2_O_2_. (Ai) Pseudo-colour images (green) of Ca^2+^ signalling evoked by ACh (100 nM), and ACh (100 nM) in the presence of H_2_O_2_. (100 μM). Scale bar: 20 μM. (Aii) Overlaid Ca^2+^ signalling traces from ∼200 cells (shown in A) with the average shown as the black line in response to ACh and ACh+H_2_O_2_. Individual Ca^2+^ traces are coloured according to the magnitiude of the control (ACh) response. (Aiii) Rastergram plot of Ca^2+^ signals. Each red dot represents a Ca^2+^ peak in each cell (shown on the left axis) ACh (100 nM) left-side and ACh (100 nM) + H_2_O_2_ (100 μM) right-side. (Aiv) The effect of H_2_O_2_ varied on Ca^2+^ signals across cells. The panel shows traces of Ca^2+^ signalling from three typical cells from (Ai). The blue lines are the Ca^2+^ signals evoked by ACh (100 nM) and the red lines ACh (100 nM) + H_2_O_2_ (100 μM). Cell 3 was largely unaffected by H_2_O_2_. (B) Density plot of mean peak value of Ca^2+^ signalling from cells treated with ACh (100 nM) and ACh (100 nM) + H_2_O_2_ (100 μM). Individual data points have been coloured (from blue, low to red, high) according to the density (i.e. occurrence) of particular values (C) Summary of mean peak value of Ca^2+^ signalling in all cells. (D) Density plot of the frequency of Ca^2+^ signals. Individual data points have been coloured (from blue, low to red, high) according to the density (i.e. occurrence) of particular values (E) Summary of frequency of Ca^2+^ oscillations in all cells. (F) Summary of percentage of ACh-responsive cells. For all summary data (D–F) *n = 6*; *p < 0.05.

To further examine the effect of H_2_O_2_ on Ca^2+^ release from the store, the effects of the free radical were examined on ACh-evoked Ca^2+^ release. ACh (100 nM) evoked substantial Ca^2+^ signals that were heterogeneous across the endothelium and the amplitude and frequency of Ca^2+^ oscillations varied across cells. (Fig. 2A) (see also [2,38,44,49,52,55,81]). After washing out ACh, the endothelium was allowed to rest for 10 min and then challenged again with ACh (100 nM) and H_2_O_2_ (100 μM) applied simultaneously. H_2_O_2_ suppressed several aspects of ACh-evoked Ca^2+^ signalling. There was a reduction in the percentage of cells responding to ACh, a decrease in the amplitude, and a reduction frequency of oscillations in the presence of H_2_O_2_ when compared to controls (ACh alone; Fig. 2B-F). The effect of H_2_O_2_ on endothelial cells was also heterogeneous, and the free radical affected the Ca^2+^ response of some cells more than others’ (see Fig. 2Aiv). In the absence of H_2_O_2_, ACh (100 nM; 10 min. apart) evoked reproducible Ca^2+^ signals (Figure S1).

**Fig. 3:**
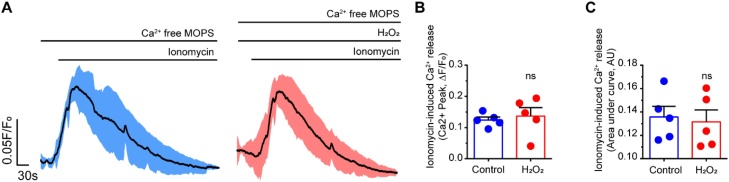
Internal Ca^2+^ store content was unchanged in H_2_O_2_. (A) The Ca^2+^ store content was assessed using ionomycin (2 μM) applied in a Ca^2+^-free PSS. The left panel shows the averaged ionomycin-induced Ca^2+^ transient in the absence of H_2_O_2_ (100 μM) while the right panel is in the presence of H_2_O_2_. The black lines is the mean of five independent preparations blue and red lines show the standard error of the mean (SEM). (B–C) Summary data of mean peak response (B) and area under the curve (C) of ionomycin-induced Ca^2+^ increase *(n = 5*).

To determine if the internal Ca^2+^ store content was altered by H_2_O_2_, ionomycin (2 μM, in Ca^2+^ free PSS) was applied in the absence or presence of H_2_O_2_ (100 μM). Ionomycin-evoked Ca^2+^ signals were not significantly altered by H_2_O_2_ (Fig. 3A). Two measurements were used in this analysis; the amplitude of ionomycin-induced Ca^2+^ release and the area under the curve (Fig. 3B, C). Each measure was unchanged suggesting that H_2_O_2_ did not deplete the internal Ca^2+^ store.

**Fig. 4:**
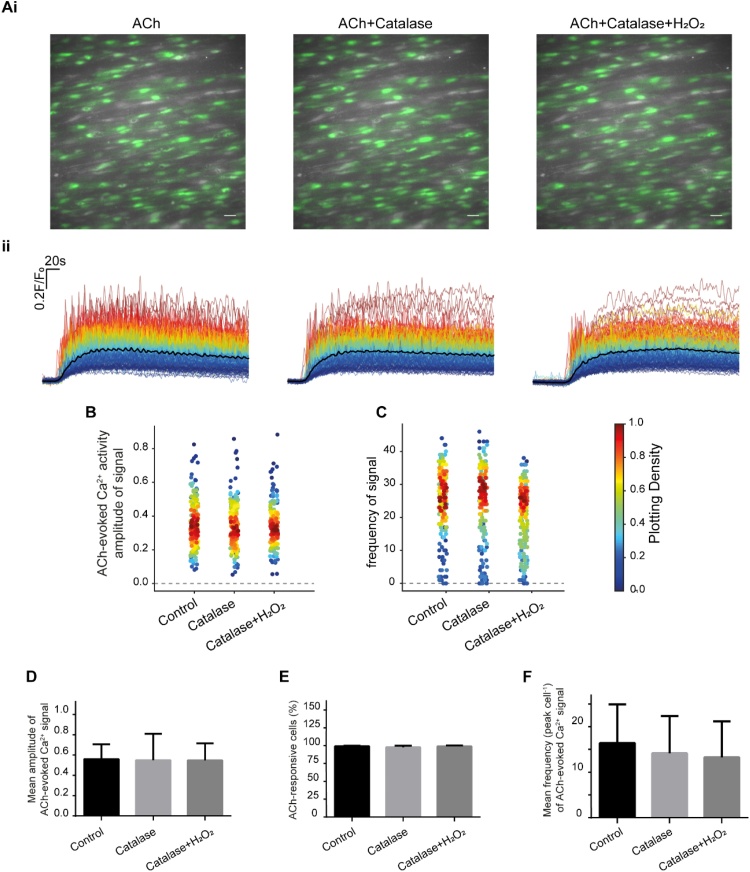
Catalase eliminated of the effect of H_2_O_2_ on ACh-evoked Ca^2+^ signalling. (Ai) Pseudo colour (green) images of Ca^2+^ signalling evoked by ACh (100 nM), ACh (100 nM) + Catalase (1000 U/ml) and ACh (100 nM) + Catalase (1000 U/ml) + H_2_O_2_ (100 μM). Scale bar: 20 μM. (Aii) Overlaid Ca^2+^ signalling traces from each endothelial cell with treatment of ACh (100 nM), ACh (100 nM) + Catalase (1000 U/ml) and ACh (100 nM) + Catalase (1000 U/ml) + H_2_O_2_ (100 μM). Individual Ca^2+^ traces are coloured according to the magnitiude of the control (ACh) response. (B) Density plot of mean peak value of Ca^2+^ signalling. Individual data points have been coloured (from blue, low to red, high) according to the density (i.e. occurrence) of particular values (C) Density plot of number of the frequency Ca^2+^ signalling. (D) Summary data showing mean peak value of Ca^2+^ signalling in all cells. (E) Summary data showing the percentage of active cells. (F) Summary data showing the frequency of Ca^2+^ signals in all cells (*n = 6* for all summary data).

To confirm that the suppression of ACh-evoked Ca^2+^ signalling in endothelial cells arose from H_2_O_2_, catalase-peg (1000 U/ml) was used to breakdown H_2_O_2_. Catalase by itself did not alter the Ca^2+^ signal evoked by ACh (100 nM) when compared to controls (Fig. 4A-F). Furthermore, in the presence of catalase, H_2_O_2_ (100 μM) did not alter the amplitude (Fig. 4B, D), or the frequency (Fig. 4C,F) of the ACh-evoked Ca^2+^ signals, nor did it alter the percentage of cells activated by ACh (Fig. 4E). These data suggest that H_2_O_2_ supresses ACh-evoked Ca^2+^ signals in native endothelial cells.

**Fig. 5:**
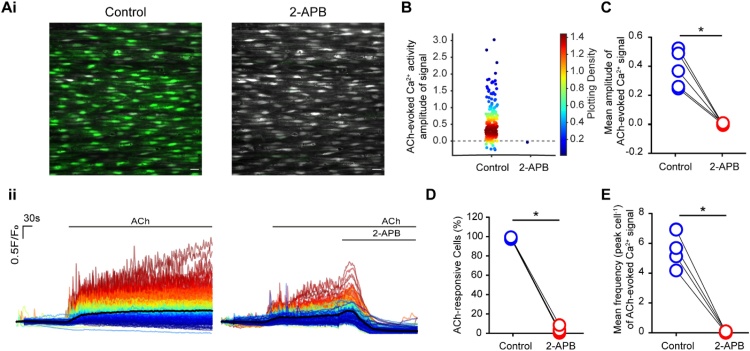
2-APB inhibits ACh-evoked Ca^2+^ signalling. (Ai) Pseudo colour (green) images of Ca^2+^ signals evoked by ACh (100 nM) and ACh (100 nM) with 2-APB (100 μM). Scale bar: 20 μM. (Aii) Overlaid Ca^2+^ signalling traces of ACh (100 nM; left) and ACh (100 nM) with 2-APB (100 μM; right). Individual Ca^2+^ traces are coloured according to the magnitiude of the control (ACh) response. (B) Density plot of mean peak value of Ca^2+^ signalling. Individual data points have been coloured (from blue, low to red, high) according to the density (i.e. occurrence) of particular values. (C) Summary data of the mean peak value of Ca^2+^ signalling (E) percentage of active cells and (D) number of peaks of Ca^2+^ signalling. For all summary data (D–E), *n = 5*, * *p<*0.05.

In native endothelial cells, ACh-evoked Ca^2+^ release requires activation of IP_3_Rs [1]. In support, ACh-evoked Ca^2+^ release was rapidly blocked by 2-APB (Fig. 5A-F). 2-APB significantly attenuated the amplitude (97% reduction; Fig. 5A, B, C) and frequency (99% reduction; Fig. 5E) of ACh-evoked Ca^2+^ signals, and the percentage of active cells activated by ACh (97% reduction; Fig. 5D).

**Fig. 6:**
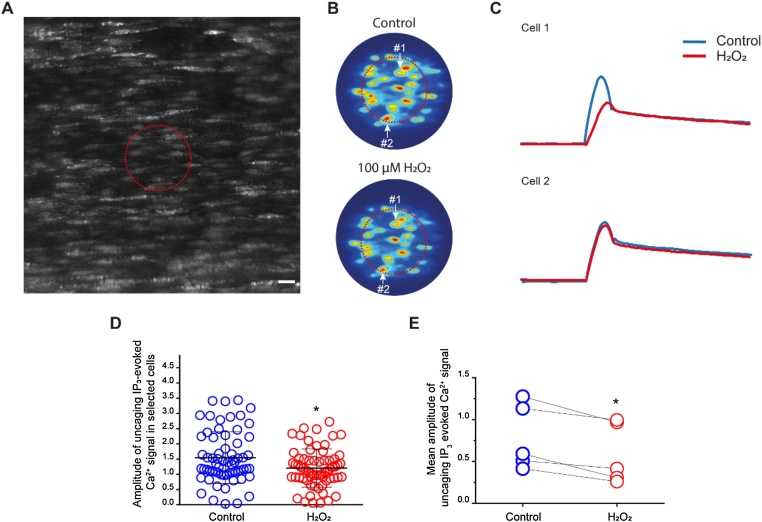
H_2_O_2_ suppresses IP_3_ receptor activity. (A) Representative image showing endothelial cells of an intact artery. The red circle demarks the area that was selected for photolysis of caged IP_3_. Scale bar: 20 μM. (B) Heat map of Ca^2+^ signalling stimulated by photolysis of caged IP_3_ in the preselected area. (C) Uncaging IP_3_-evoked Ca^2+^ signalling traces of two representative single cells (cell 1 and cell 2 from B) before and after 20 min incubation of H_2_O_2_ (100 μM). As with ACh there was heterogeneity in the responses of cells to H_2_O_2_. The peak value of the Ca^2+^ signal was either unaltered (cell 2) or suppressed (cell 1) by H_2_O_2_. (D) Summary data showing the Ca^2+^ signal peak after uncaging. Control: blue; H_2_O_2_ (100 μM): red (*n = 5*). (E) Averaged peak value of Ca^2+^ signals from five different experiments. Control: blue; H_2_O_2_ treated: red, (*n = 5*; *p < 0.05).

To determine which part of the IP_3_ pathway Ca^2+^ release was modified by H_2_O_2_, we performed experiments using the membrane-permeant, photoactivateable form of IP_3_ (caged-IP_3_5 μM). IP_3_, released via the photolysis of caged-IP_3_, directly activates IP_3_Rs [13,51] and evoked a Ca^2+^ response (Fig. 6A-E). H_2_O_2_ (100 μM) significantly attenuated the Ca^2+^ response to photolysis of caged-IP_3_ (22% reduction; Fig. 6C-E). Again, there was heterogeneity in the sensitivity to H_2_O_2_ and some cells were less affected than others (Fig. 6C). These results demonstrate that H_2_O_2_ reduces ACh-evoked Ca^2+^ release by altering either the activity of IP_3_ receptors or the interaction between IP_3_ and IP_3_Rs.

**Fig. 7:**
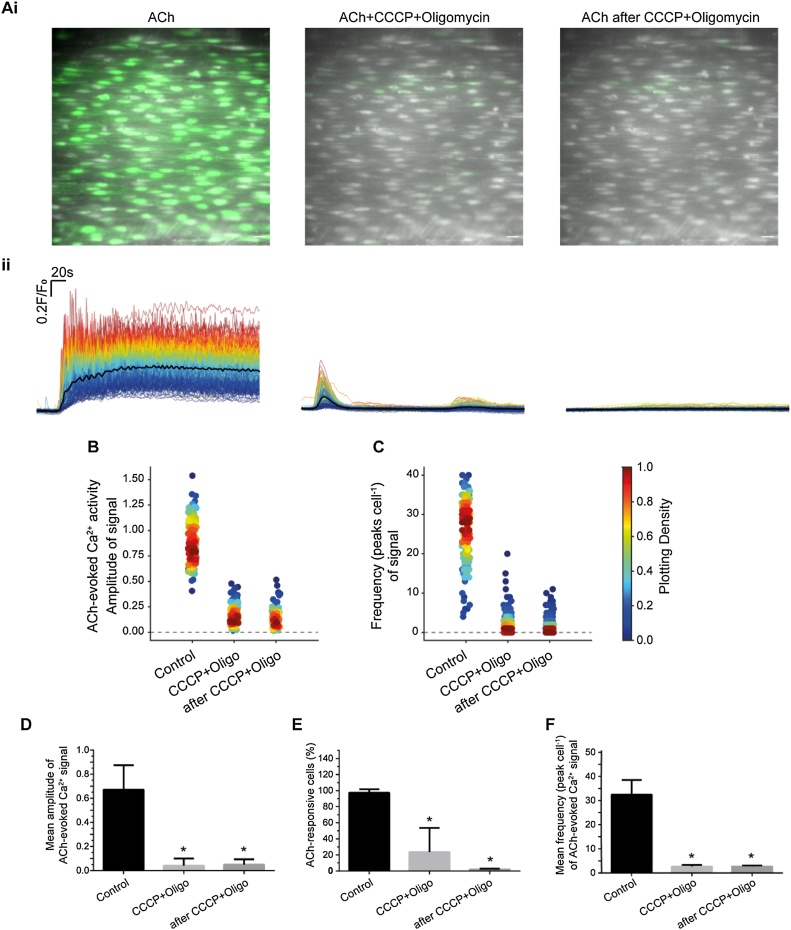
The uncoupler, CCCP, and ATP synthase blocker, oligomycin, inhibited ACh-evoked Ca^2+^ signals. (Ai) Pseudo colour (green) images of Ca^2+^ activity evoked by ACh (100 nM; left), ACh (100 nM) + CCCP (5 μM) + oligomycin (6 μM; middle), and after washout of the CCCP and oligomycin (right). Scale bar: 20 μM. (Aii) Overlaid single cell Ca^2+^ traces from the endothelial cells shown in Aii. Individual Ca^2+^ traces are coloured according to the magnitiude of the control (ACh) response. (B) Density plot of mean peak value of the Ca^2+^ signal. Individual data points have been coloured (from blue, low to red, high) according to the density (i.e. occurrence) of particular values (C) Density plot of the frequency of Ca^2+^ signals. (D) Summary of the mean peak value of Ca^2+^ signals in all cells, (E) Percentage of active cells (F) and the frequency of signals in all cells. For all summary data (D–F), *n = 3*, *p<0.05.

**Fig. 8:**
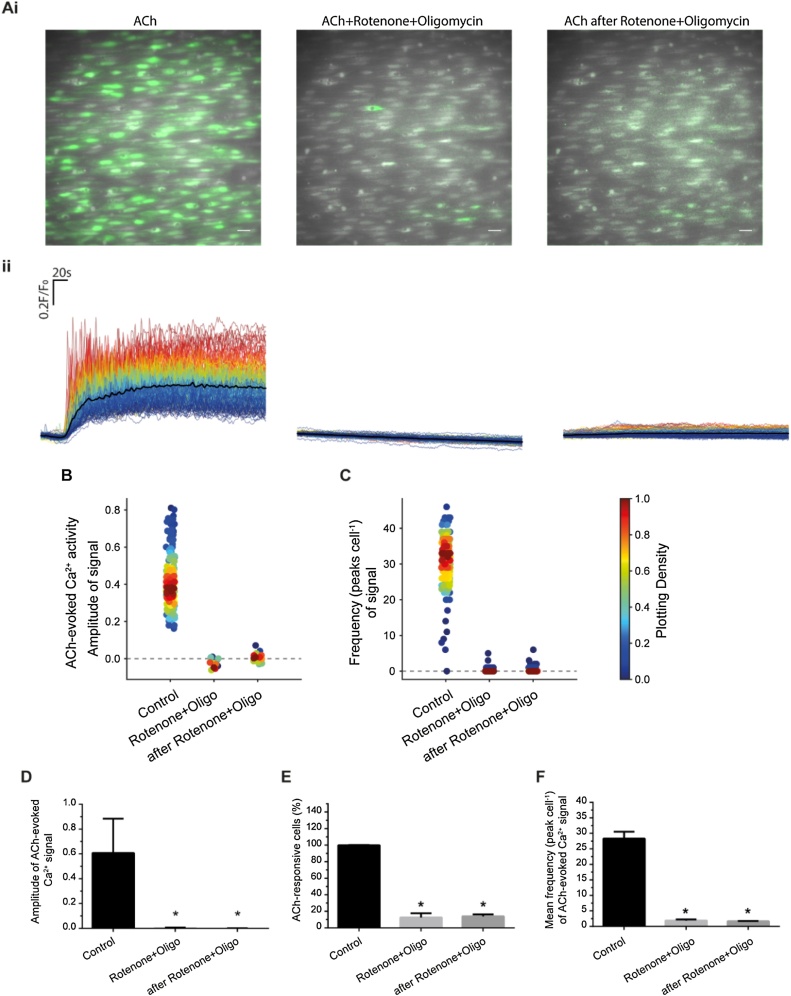
Inhibition of complex I blocked ACh-evoked Ca^2+^ signalling. (Ai) Pseudo colour (green) images of Ca^2+^ signals evoked by ACh (100 nM), ACh (100 nM) + Rotenone (2 μM) + oligomycin (6 μM) and after washout of rotenone and oligomycin. Scale bar: 20 μM. (Aii) Overlaid Ca^2+^ signalling traces, from the cells shown in Ai, evoked by ACh (100 nM), ACh (100 nM) + Rotenone (2 μM) + oligomycin (6 μM) and after washout of rotenone and oligomycin. (B) Density plot of mean peak value of Ca^2+^ signalling. Individual data points have been coloured (from blue, low to red, high) according to the density (i.e. occurrence) of particular values (C) Density plot of number of peaks of Ca^2+^ signalling. (D) Summary of the mean peak value of Ca^2+^ signals in all cells, (E) Percentage of active cells (F) and the frequency of signals in all cells. For all summary data (D–F), *n = 3*, *p<0.05.

Since H_2_O_2_ is reported to increase the activity of IP_3_Rs [11], the question arises as to how H_2_O_2_ is able to decrease IP_3_-evoked Ca^2+^ release. Mitochondria exert profound control of IP_3_-evoked Ca^2+^ release [58,80] and H_2_O_2_ has been shown to alter mitochondrial function [56]. These observations raise the possibility that H_2_O_2_ may exert effects on IP_3_R indirectly. To determine if mitochondria mediate the effects of H_2_O_2_, we investigated the effect of uncoupling mitochondria on Ca^2+^ release from the internal Ca^2+^ store. To do this, the uncoupler, CCCP, and the complex I inhibitor, rotenone, were used in separate experiments. Each drug was used in combination with the ATP synthase inhibitor oligomycin, to prevent reversal of the ATP synthase. CCCP (5 μM) and oligomycin (6 μM) inhibited ACh-evoked Ca^2+^ signalling (Fig. 7A, B & Figure S2); the inhibition remained even after CCCP and oligomycin wash out (Fig. 7A, B). CCCP and oligomycin significantly reduced the amplitude and frequency of ACh-evoked Ca^2+^ signals, and the percentage of cells activated by ACh (Fig. 7C-F). Similarly, rotenone (2 μM) and oligomycin (6 μM) also inhibited ACh-evoked Ca^2+^ signaling (Fig. 8A-F). These results demonstrate that mitochondria regulate IP_3_-mediated Ca^2+^ release and that mitochondrial membrane potential depolarization inhibits IP_3_-evoked Ca^2+^ release.

**Fig. 9:**
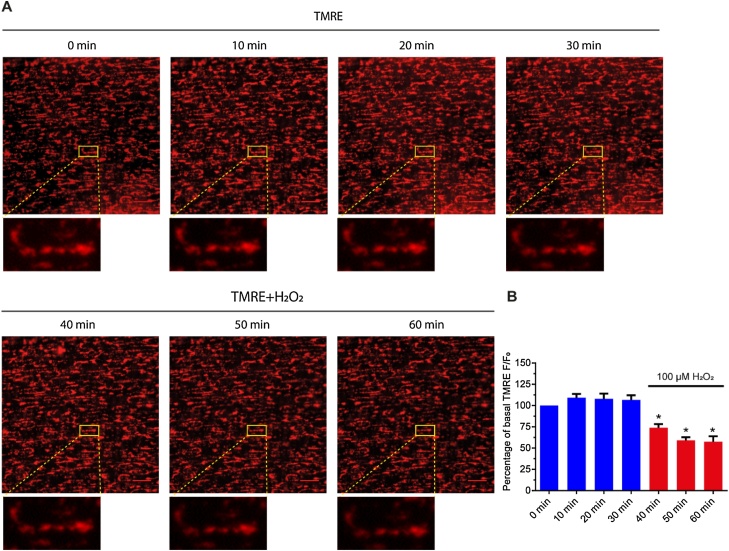
Depolarisation of mitochondria by H_2_O_2_. (A) Pseudo colour images (red) of the mitochondrial membrane potential (reported by the membrane potential sensitive dye TMRE). In control (0 min ∼ 30 min) and after H_2_O_2_ (100 μM; 30 min–60 min). H_2_O_2_ decrease the mitochondrial membrane potential as revealed by the decrease in mitochondrial TMRE fluorescence intensity. Scale bar: 20 μM. (B) Quantification of the normalized intensity of TMRE (*n = 6*). *p < 0.05.

To explore the role H_2_O_2_ plays in mitochondria-regulated Ca^2+^ signaling, mitochondrial membrane potential was assessed using TMRE. TMRE is a lipophilic cation that is rapidly sequestered by the negatively-charged (∼−180 mV) mitochondrial membrane potential [21]. TMRE was imaged for 30 min to ensure the stability of the indicator. After 30 min, H_2_O_2_ (100 μM) was introduced and TMRE imaged for a further 30 min. H_2_O_2_ caused a significant decrease in TMRE fluorescence intensity (Fig. 9A, B). These findings suggest that H_2_O_2_ may suppress IP_3_-evoked Ca^2+^ release by depolarizing mitochondria. In a control experiments, to confirm mitochondrial localization, TMRE (60 nM) was loaded together with mitotracker green (100 nM). The two mitochondrial indicators largely overlapped in their localization (Figure S3). As expected from a mitochondrial localization of the dyes, mitochondrial membrane potential depolarization with CCCP (5 μM; applied with oligomycin (6 μM)) dispersed punctuate TMRE staining and reduced mitotracker green labelling (Figure S3).

## 4 Discussion

**Fig. 10:**
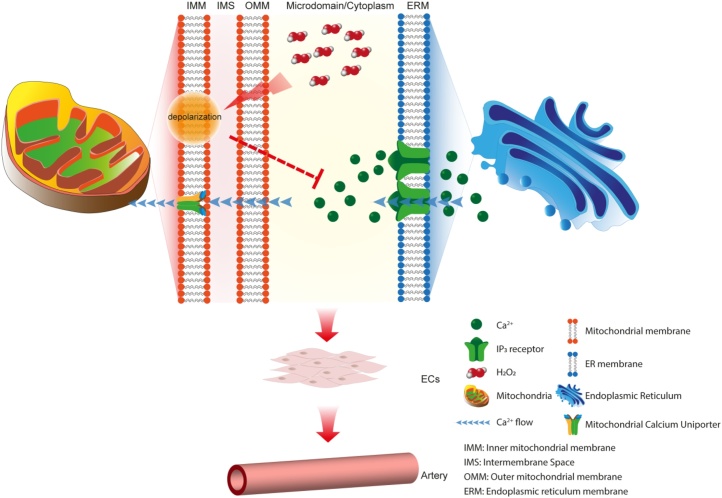
Proposed mechanism for mitochondria regulation Ca^2+^ signalling in endothelial cells. The mitochondrial membrane potential is depolarized by H_2_O_2_. The depolarized membrane potential limits the driving force for Ca^2+^ uptake by mitochondria. As result, the concentration of Ca^2+^ in microdomain area between mitochondria and endoplasmic reticulum is increased and the ion suppresses Ca^2+^ release through IP_3_ receptor from the endoplasmic reticulum. Limited Ca^2+^ endothelial and artery function. On the other hand, reduced uptake of Ca^2+^ into mitochondria will lead to less production of ATP, therefore, reduced the metabolic production of H_2_O_2_. The negative feedback loop may help endothelial cells maintain normal metabolism and cell survival.

Interaction between the internal Ca^2+^ store and mitochondrial are critical in regulating cell signalling and cell performance. Several diffusible mediators communicate between the two organelles to control cell and tissue function. Of these, Ca^2+^ and H_2_O_2_ are of particular significance. Mitochondria are a major source, and the internal Ca^2+^ store a target, for H_2_O_2_. H_2_O_2_ modulates Ca^2+^ transport mechanisms on the internal Ca^2+^ store [5,12,24,60] and in turn, Ca^2+^ release from the store modulates mitochondrial function by regulating the enzymes of the Krebs cycle and oxidative phosphorylation [41]. Ca^2+^-induced changes in metabolic rate result in altered oxygen consumption, respiratory chain electron leakage and H_2_O_2_ levels [14]. Here, we have demonstrated H_2_O_2_ depolarises mitochondria and inhibits spontaneous and agonist-evoked IP_3_-induced Ca^2+^ signals. We suggest that suppression of IP_3_-evoked Ca^2+^ release arises from a H_2_O_2_-induced the decrease in mitochondrial membrane potential (Fig. 10).

Mitochondria are potent modulators of IP_3_-evoked Ca^2+^ release. Ca^2+^ uptake by mitochondria may promote Ca^2+^ release from IP_3_Rs [18,19,23,43,54,65,72,73,78], limit IP_3_-evoked Ca^2+^ signals [4,34] or slow IP_3_-evoked Ca^2+^ wave progression [10,15,32,61,69,84]. At least two mechanisms have been proposed to account for mitochondrial control of IP_3_R activity. First, at sites of close contact between the internal Ca^2+^ store and mitochondria [26,48], channels on the internal Ca^2+^ store and mitochondrial channels (e.g. the uniporter and voltage-dependent anion-selective channel) may cluster, and Ca^2+^ uptake into mitochondria occurs at these sites [25,33,63,64]. Ca^2+^ uptake depends critically on the mitochondrial membrane potential. As the membrane potential decreases, so does mitochondrial Ca^2+^ uptake. Mitochondrial Ca^2+^ uptake limits a negative feedback process that operates at IP_3_ receptors to maintain Ca^2+^ release [19,54]. In smooth muscle, mitochondrial Ca^2+^ uptake is fast enough to regulate local spontaneous Ca^2+^ signals arising from IP_3_Rs (Ca^2+^ puffs) [57] and regulates store-operated Ca^2+^ entry [59], demonstrating tight functional coupling between IP_3_Rs and mitochondria. However, in other studies, close coupling between the internal store and mitochondria was not required for mitochondrial control of Ca^2+^ release to occur [80]. In the second mechanism proposed to account for mitochondrial control of the internal store, mitochondrial ATP production modulates Ca^2+^ release. When ATP production was restricted Ca^2+^ release was inhibited. This mechanism permits mitochondria to control Ca^2+^ release while being positioned far from the internal Ca^2+^ store [80]. Several studies show that ATP maintains IP_3_-mediated Ca^2+^ release. ATP potentiates IP_3_-induced Ca^2+^ release in permeabilized cells and from native endoplasmic reticulum vesicles, and it enhances activation of IP_3_-gated channels and purified, reconstituted IP_3_Rs [28,39,50,66] by increasing the open time of the channel s[7]. Thus factors provided by mitochondria may diffuse to IP_3_R to maintain IP_3_R activity.

The present results highlight another control mechanism which may operate between mitochondria and the internal Ca^2+^ store i.e. diffusion of H_2_O_2_. Our results suggest that the control of IP_3_ release by H_2_O_2_ is indirect and mediated by depolarization of mitochondria so combines aspects from both proposals (diffusible substance and mitochondrial depolarization). H_2_O_2_-mediated depolarization of the mitochondrial membrane potential will decrease the driving force for Ca^2+^ uptake by mitochondria and so limit negative feedback control of IP_3_-evoked Ca^2+^ release. Several proposals exist for the mechanisms by which H_2_O_2_ may depolarize mitochondria [30,31] and include inhibition of alpha-ketoglutarate dehydrogenase [22,56] or succinate dehydrogenase [56], or activation of the permeability transition pore [83]. Interestingly, the effect of H_2_O_2_ was not homogeneous across all cells and some were affected more than others. The reason for the heterogeneity is not clear, but perhaps differences in basal levels of H_2_O_2_, or metabolic state of the cells may contribute. Alternatively, antioxidant enzymes whose activities are directed at reducing hydrogen peroxide, such as catalase, glutathione peroxidase, and thioredoxin peroxidase, may vary across cells.

While the present results suggest that H_2_O_2_ inhibition of IP_3_R is indirect and mediated via mitochondria, ROS may also directly alter IP_3_ receptor activity. However, H_2_O_2_ is often reported to promote rather than inhibit IP_3_R activity. When H_2_O_2_ transients were prevented, Ca^2+^ oscillations were inhibited in some cells, implying that H_2_O_2_ makes subsequent Ca^2+^ release more likely to occur [11]. In support of these observations, in various cultured endothelial cell lines (HAECs, LECs, HUVECs and CVECs), H_2_O_2_ induces Ca^2+^ release from the internal store [27,37,77]. Various exogenously added oxidants stimulate rather than inhibit IP_3_R-mediated Ca^2+^release. Thimerosal [12,16,42], t-butylhydroperoxide [9], and diamide [45,46] each increase IP_3_-evoked Ca^2+^ release. In the case of thimerosal, the proposed mechanism involves an increased sensitivity of the receptor to [IP_3_] that results in Ca^2+^oscillations occurring at basal [IP_3_] in unstimulated cells [5,40]. Although sensitization to IP_3_may be a general mechanism responsible for the action of other oxidants, it has also been suggested that they (oxidants) may alter the sensitivity of IP_3_R to Ca^2+^ [9,45]. Our results suggest that the indirect effect of H_2_O_2_ on endothelial mitochondria may dominate control of IP_3_R. Since H_2_O_2_ may *increase* IP_3_R activity, these findings buttress the proposal that inhibition of Ca^2+^ release by H_2_O_2_ is indirect.

H_2_O_2_ regulates several key modulators of cell activity including cell proliferation, migration, and differentiation, and because H_2_O_2_ is membrane permeable, the ROS may exert widespread control across many endothelial cells to regulate cardiovascular function. H_2_O_2_ mediates at least part of its effects through changes in Ca^2+^ signalling. Ca^2+^ influx in native and in cultured endothelial cell lines is evoked by H_2_O_2_. In the native endothelium of superior epigastric arteries, H_2_O_2_ evoked Ca^2+^ increases result from Ca^2+^ influx via TRPV4 channels [67]. In mouse and human lung microvascular endothelial cell lines (MLMVEC, HPAE, H5V and HLMVEC) H_2_O_2_ evokes Ca^2+^ influx via TRPV4 or TRPM2 [70,71]. Our results reveal an additional complexity in the effects of H_2_O_2_. H_2_O_2_ may inhibit IP_3_-evoked Ca^2+^ release in native endothelial cells. The inhibition of IP_3_ evoked Ca^2+^ by H_2_O_2_ may be an indirect and mediated via depolarization of the mitochondrial membrane potential. This process may serve as a negative feedback modulation of mitochondrial function. Ca^2+^ increases associated with cell activity may initially stimulate mitochondrial ATP production. However, an increase in electron transport activity will result in increased ROS production, with a decrease in mitochondrial membrane potential and inhibition of IP_3_-evoked Ca^2+^ release as a result. H⁠_2_O⁠_2_ mediated mitochondrial depolarization may be a mechanism by which the ogranelles inhibit IP⁠_3_-evoked Ca^2+^ signalling to protect themselves against Ca^⁠2+^ overload .

## Author contributions

XZ, MDL,CW & JGM developed the concept. XZ performed the experiments. JGM & XZ drafted the manuscript. JGM, ZX, CW & MDL edited and revised the manuscript. CW & JGM sourced funding. All authors approved the final version of the manuscript.

## Declaration of Competing Interest

None.
