# Supplementary material for: Hydrogen peroxide depolarizes mitochondria and inhibits IP3-evoked Ca2+ release in the endothelium of intact arteries
Source: Cell Calcium. 2019 Dec;84:102108. doi: 10.1016/j.ceca.2019.102108 (PMC6891240; doi:10.1016/j.ceca.2019.102108)
Supplement: Supplementary file 1 [file mmc1.docx]

**SUPPLEMENTARY DATA**


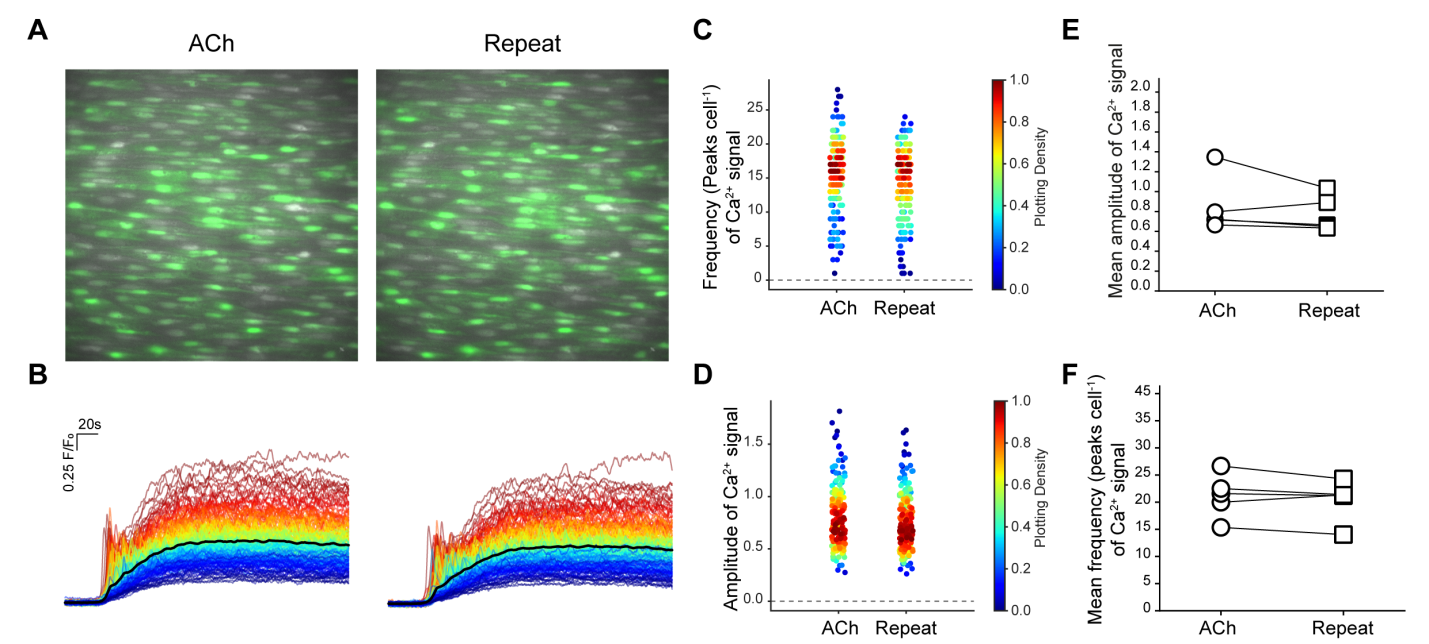


**Figure S1. Reproducible ACh evoked Ca^2+^ signals.** (A) Pseudo-colour images (green) of Ca^2+^ signalling evoked by ACh (100 nM) applied twice. Scale bar: 20 μM. (B) Overlaid Ca^2+^ signalling traces from ~200 cells (shown in A) with the average shown as the black line. (C) Density plot of the frequency of Ca^2+^ signals to the two applications of ACh (100 nM). Individual data points have been coloured (from blue, low to red, high) according to the density (i.e. occurrence) of particular values. (D) Density plot of mean peak amplitude of Ca^2+^ signalling from cells treated with ACh (100 nM). Individual data points have been coloured (from blue, low to red, high) according to the density (i.e. occurrence) of particular values. (E) Mean peak value of Ca^2+^ signalling in all cells. (F) Mean frequency of Ca^2+^ oscillations in all cells (*n=5*).


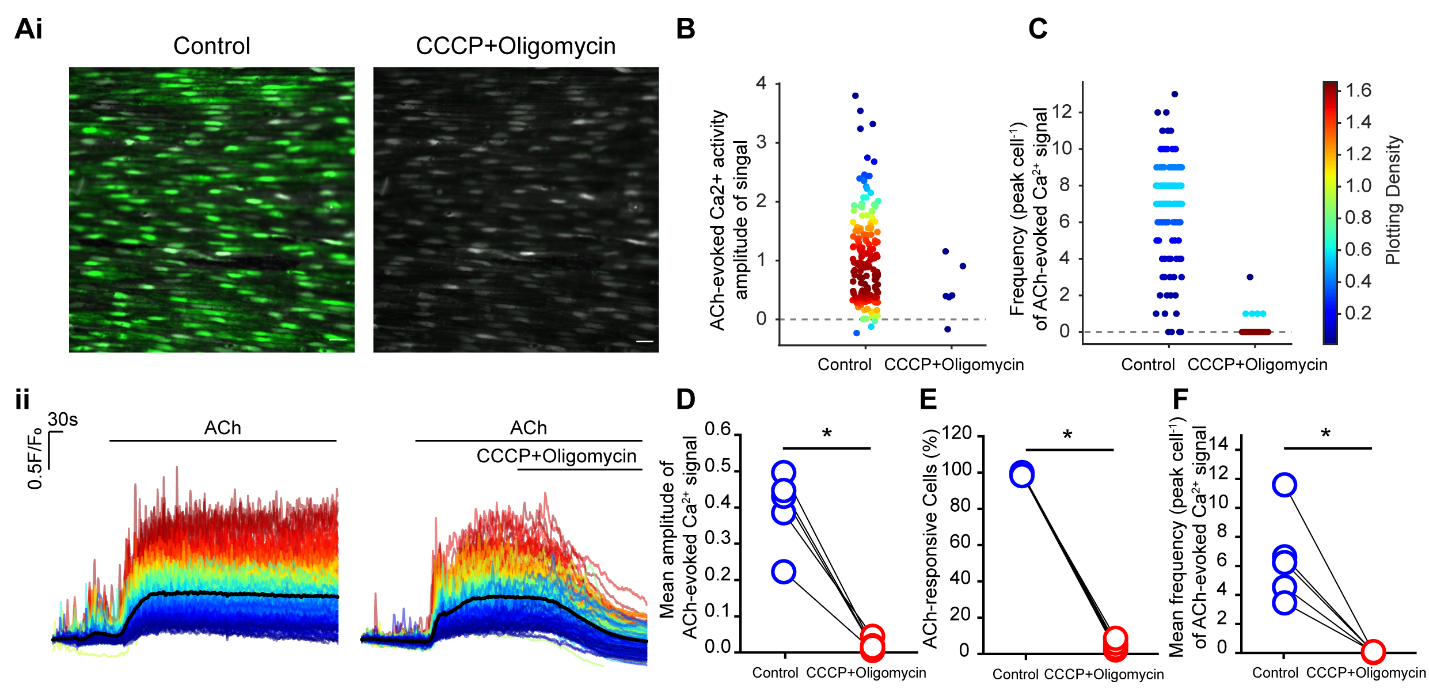


**Figure S2. CCCP rapidly inhibits ACh-evoked Ca^2+^ signals.** (Ai) Pseudo colour (green) image of the Ca^2+^ response evoked by ACh (100 nM; left panel) and ACh (100 nM) + CCCP (5 μM) + oligomycin (6 μM; right panel). Scale bar: 20 μm. (Aii) Overlaid Ca^2+^ signal traces from each of the ~200 cells (shown in A) during 10 min recording with treatment of ACh (100 nM; left panel) and ACh (100 nM) + CCCP (5 μM) + oligomycin (6 μM; right panel). CCCP and oligiomycin rapidly inhibited the ACh-evoked Ca^2+^ increase. (B) Density plot of mean peak value of the Ca^2+^ signal. Individual data points have been coloured (from blue, low to red, high) according to the density (i.e. occurrence) of particular values (C) Density plot of the frequency of Ca^2+^ signals. (D) Summary of the mean peak value of Ca^2+^ signals in all cells, (E) Percentage of active cells (F) and the frequency of signals in all cells in the absence and presence of CCCP and oligiomycin. For all summary data (D-F), *n=5*, **p<*0.05.


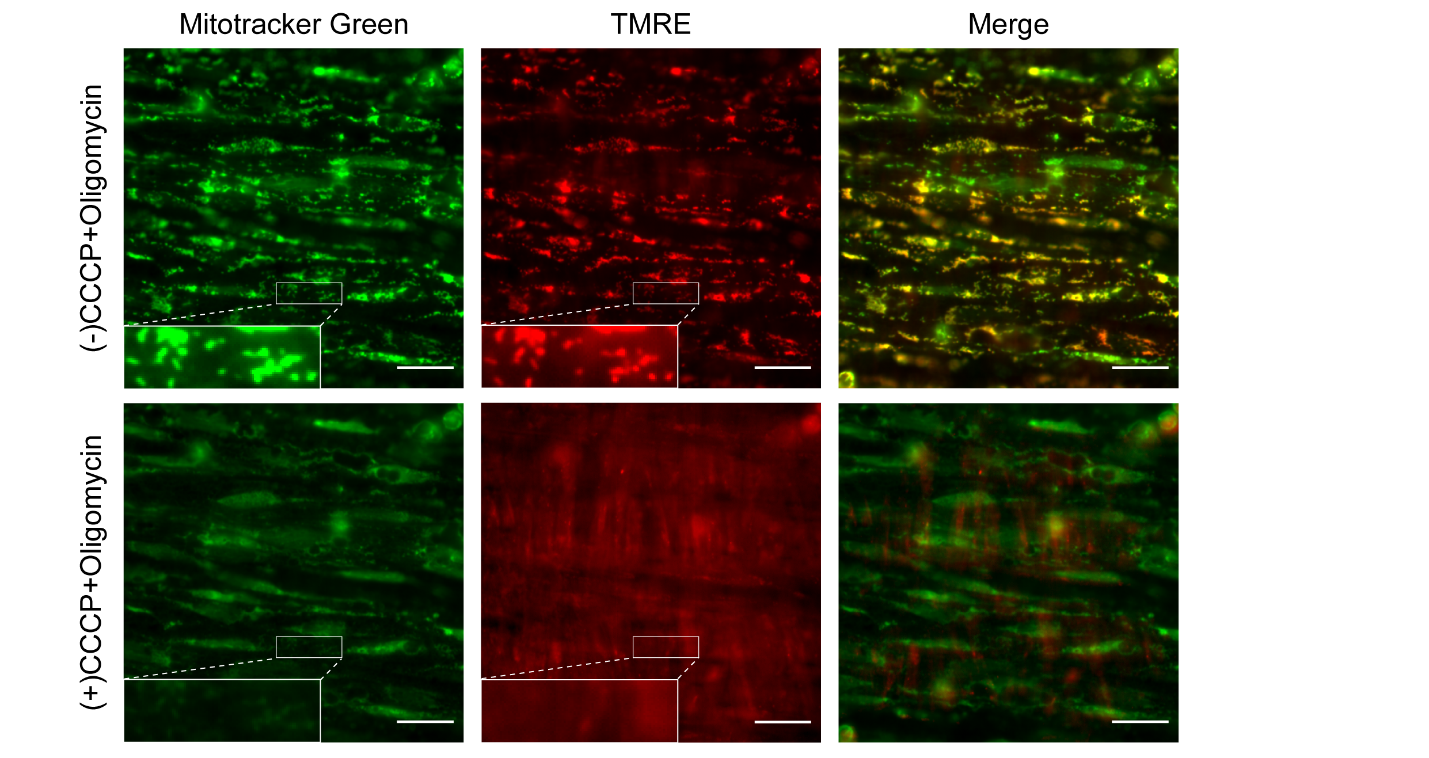


**Figure S3.** **The uncoupler CCCP (applied with the ATP synthase blocker oligomycin) collapses the mitochondrial membrane potential.** Endothelial cells of the intact artery were labelled with two mitochondrial membrane markers, mitotracker green (100 nM; left) and TMRE (60 nM; middle) and a merge of both (right). The upper panel shown mitochondrial localization and structure before CCCP (5 μM) and oligomycin (6 μM). The lower panel shown shows mitochondria after collapse of the mitochondrial membrane potential with 5 min treatment of CCCP and oligomycin. Mitotracker green staining remained though diminished while TMRE staining was completely dispersed as expected from loss of the dye in mitochondria. Scale bar :20 μM.
